# Biomaterials based on noncovalent interactions of small molecules

**DOI:** 10.17179/excli2020-2656

**Published:** 2020-08-05

**Authors:** Jiaqi Guo, Changhao Tian, Bing Xu

**Affiliations:** 1 Department of Chemistry, Brandeis University, 415 South St., Waltham, MA 02453, USA; 2 Department of Physics, Nanjing University, 22 Hankou Road, Nanjing, Jiangsu, 210093, China

**Keywords:** noncovalent interactions, supramolecular biomaterials, self-assembly, enzymes, application

## Abstract

Unlike conventional materials that covalent bonds connecting atoms as the major force to hold the materials together, supramolecular biomaterials rely on noncovalent intermolecular interactions to assemble. The reversibility and biocompatibility of supramolecular biomaterials render them with diverse range of functions and lead to rapid development in the past two decades. This review focuses on the noncovalent and enzymatic control of supramolecular biomaterials, with the introduction to various triggering mechanism to initiate self-assembly. Representative applications of supramolecular biomaterials are highlighted in four categories: tissue engineering, cancer therapy, drug delivery, and molecular imaging. By introducing various applications, we intend to show enzymatic control and noncovalent interactions as a powerful tool for achieving spatiotemporal control of biomaterials both *in* *vitro* and *in vivo* for biomedicine.

## Introduction

Covalent and noncovalent interactions are the two main categories of forces that hold atoms together. Unlike relatively strong covalent bonds that arise from sharing electrons, noncovalent interactions consist of weaker forces, such as ionic bonds, hydrogen bonds, Van der Waals interactions, and hydrophobic interactions. Noncovalent interactions, conferring reversibility to macromolecules, drive the formation of various cellular compartments and participate in numerous biological processes. Proteins, requiring correct folding to function, are the ultimate examples that rely significantly on noncovalent interactions. Another important consequence of noncovalent interactions is self-assembly. Self-assembly is a thermodynamic process that minimizes the system energy through intermolecular noncovalent interactions, generating well-defined (or ordered) nanostructures, including the characteristic nanofibers, along with nanofilaments, nanodiscs, nanorods, and nanoribbons, etc (Aggeli et al., 2001[1]; Yang et al., 2006[106]). Thus, noncovalent interactions are an inherent feature of life. Moreover, noncovalent interactions are dynamic. For example, the association and dissociation of ADP and ATP with actins modulate the dynamic equilibrium between globular actin (G-actin) and filamentous actin (F-actin), regulating cellular functions such as endocytosis, mitosis, mitochondria dynamics, and cell migration (Bugyi and Carlier, 2010[6]). The supramolecular polymerization of α-tubulins and β-tubulins leads to the formation of microtubules, which are the essential components of centrosomes, spindle fibers, cilia, and flagella (Mclntosh et al., 2018[64]). Not surprisingly, these assembly and disassembly processes involve enzymatic reactions. Being highly specific and efficient, enzymes are unique biomacromolecules that catalyze fundamental chemical reactions, like respiration and photosynthesis (Atkinson, 1969[4]). The omnipresent enzymes and continuously ongoing enzymatic reactions represent another attribute of life. 

Previous studies have focused on the final structures by self-assembly or the reaction products via breaking or forming covalent bonds by enzymatic covalent synthesis (Lewis et al., 2011[48]; Vaissier Welborn and Head-Gordon, 2018[77]). Recent development in molecular biology and cell biology reveal the significance of enzymatic supramolecular (i.e., noncovalent) assemblies for cell functions. Such insights have led to the exploration of enzymatic noncovalent synthesis, a process that incorporates both enzymatic covalent synthesis and noncovalent assemblies to form higher-order structures (Shy et al., 2019[73]). Enzymatic noncovalent synthesis usually starts from a less ordered system where the precursors undergo enzymatic reactions (e.g., enzymatic cleavage) to initiate self-assembly to result in morphological transformation (Yang and Xu, 2006[107]; Yang et al., 2004[104]). One usual result of enzymatic noncovalent synthesis of small molecules is the formation of molecular nanofibers. The entangling nanofibers form networks that trap water inside, altering rheological properties of the solution to generate hydrogels. Therefore, hydrogelation or phase transition is a good indication of self-assembly in water resulted from enzymatic noncovalent synthesis.

This review provides a brief introduction of the supramolecular biomaterials made of small molecules (especially peptides and peptide derivatives) because the ordered structures of molecular assemblies provide a versatile platform for designing emergent functions. We start with introducing common triggering mechanisms to initiate self-assembly. By discussing representative examples of supramolecular biomaterials formed intercellularly, peri/intracellularly, or subcellularly, we emphasize the applications of supramolecular biomaterials in the biomedicine, including tissue engineering, cancer therapy, drug delivery, and molecular imaging. Since a few comprehensive reviews of the related subjects already have been published (Du et al., 2015[17]; Zhou et al., 2017[124]), we mainly focus on representative examples in the past five years. Polymer supramolecular assemblies will not be included because of considerable disorders associated with conventional polymers. 

## Triggers for Making Non-Covalent Supramolecular Biomaterials

To design biomaterials that detect or respond to subtle changes in biological processes, various triggering mechanisms are useful to establish noncovalent interactions among molecules. Upon initiation, the increased noncovalent interactions result in self-assembly and eventually lead to the formation of supramolecular materials with higher organizational orders and emergent functions. Two main categories of the triggers are: (i) physicochemical changes, and (ii) enzymatic reactions.

### Physicochemical changes

**Figure 1:**
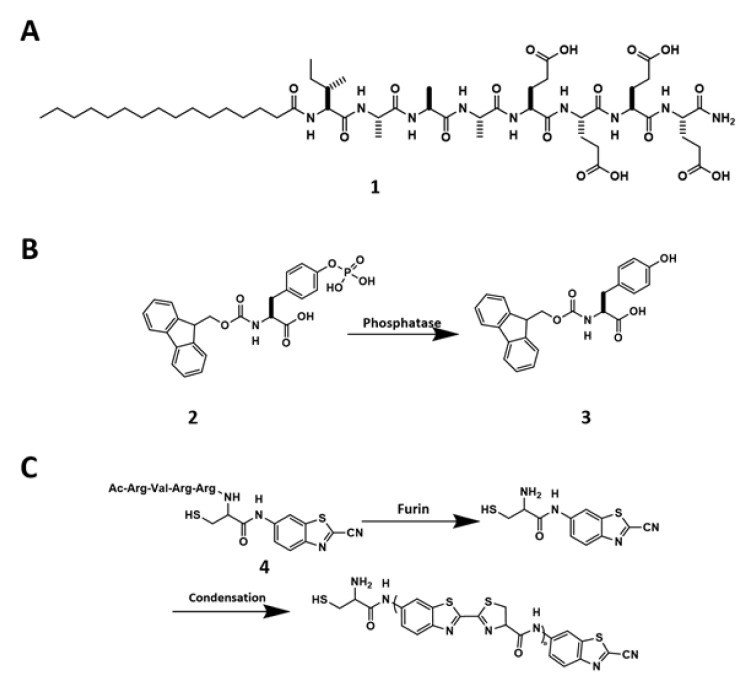
(A) Chemical structure of 1. (B) Dephosphorylation of 2 to form 3. (C) Chemical reactions of 4 that undergoes furin-guided CBT condensation.

The thermodynamics and kinetics of supramolecular interactions have to depend on temperature, leading to the development of thermal responsive materials, for example, supramolecular hydrogels (Bairi et al., 2010[5]; Roy et al., 2010[71]; Ghosh and Dey, 2009[27]). The classic “heat-cooling” method generates hydrogels via cooling homogeneous gelator solution from an elevated temperature. In this process, thermal energy at the elevated temperature drives the structural rearrangement of the molecules to eventually form a kinetically stable state after cooling. Similarly, ultrasound provides energy to break intramolecular forces, thus promoting intermolecular self-assembly to form more stable structures, which also are able to produce hydrogels. The change of pH is a convenient trigger. For example, being important parts in the peptide amphiphile, the protonable groups on the side chain of lysine, histidine, arginine, aspartic acid, and glutamic acid endow pH-responsive property to the supramolecular biomaterials made of peptides. Protonation/deprotonation results in significant changes in hydrophobicity, therefore leading to self-assembly and hydrogelation (Moyer et al., 2014[66]; Preslar et al., 2016[68]). This mechanism fits well with biological systems due to the pH spatiotemporal heterogeneity of cells. For example, Goldberger et al. reported a peptide amphiphile (**1**, Figure 1A(Fig. 1)) transforms from either isolated molecules or micelles to nanofibers upon slightly pH decrease (pH 6.6) (Ghosh et al., 2012[28]). It remains to see how this approach to be translated in the acidic tumor microenvironment. Another type of stimuli is ligand-receptor interactions, which is extensively used by nature, but less frequent in developing biomaterials. The most explored example is to re-establish hydrogen bonding and hydrophobic interactions by a ligand-receptor pair made of vancomycin and D-Ala-D-Ala (Healy et al., 2000[37]) to form a supramolecular hydrogel (Zhang et al., 2003[113]). Increased ionic strength in some cases also triggers self-assembly, as reported by Goldberger et al. (2011[29]) that 5 mM Ca^2+^ renders morphological transition to form nanofibers. In addition, introducing functional groups into precursors allow certain chemical reactions, such as hydrolysis (Zhao et al., 2011[114]), redox reactions (Chen et al., 2012[8]; Cao et al., 2012[7]), and photochemical reactions (Yamamoto et al.,1999[99]), to take place, which are useful types of triggering mechanisms for controlling intermolecular interactions and initiating self-assembly.

### Enzymatic reaction

Enzymatic reactions, being universal in the biology, are emerging as a powerful trigger for fast and specific assembling processes to generate biomaterials, including hydrogels. For example, enzymatic reactions boost the hydrophobicity of peptide precursors to generate hydrogelators, therefore initiating self-assembly processes. The first example of enzyme-instructed self-assembly (EISA) uses alkaline phosphatase (ALP) to clip off the phosphate group from the precursor (Fmoc-tyrosine phosphate, **2**) to form a hydrogelator (**3**) (Figure 1B(Fig. 1)). Fibrillar networks trap water inside to form a supramolecular hydrogel (Yang et al., 2004[104]). Different from the heat-cooling method, EISA usually gives rise to nanofibers with β-sheet structures, which are essential for receptor recognition (Shang et al., 2019[72]). More importantly, EISA is more compatible to cells than other self-assembly triggers, such heating and cooling.

Besides ALP, which has a broad spectrum of substrates and fast kinetics, other enzymes, including enterokinase (He et al., 2017[35]), carboxylesterases (CES) (Zhang et al., 2019[112]), matrix metalloproteinases (Kalafatovic et al., 2016[41]), β-galactosidase (Xu et al., 2019[97]; Akama et al., 2018[2]), and β-lactamase (Yang et al., 2007[105]), have acted as the catalysts for EISA. It is also useful to combine enzymatic reaction with fast chemical reactions for EISA. For example, furin-mediated 2-cyanobenzothiazole (CBT) condensation represents a unique strategy to trigger self-assembly (Ghosh et al., 2012[28]). Upon furin clipping off the RVRR sequence from the precursor (**4**), the exposures of both amine and thiol groups facilitate the condensation reaction with CBT, allow the condensation product to accumulate in the cancer cells that upregulate glutathione (GSH) and furin (Figure 1C(Fig. 1)). This efficient and biocompatible condensation has received extensively exploration in the field of molecular imaging (Wang et al., 2019[78]) and cancer therapy (Yuan et al., 2015[109]).

## Applications of Biomaterials Based on Noncovalent Interactions

### Nonenzymatic noncovalent biomaterials

Owing to various physicochemical triggering mechanisms, nonenzymatic noncovalent biomaterials have wide range of applications in different conditions. Firstly, noncovalent interactions, such as ligand-receptor interactions between biomolecules, offer a versatile way to create biomaterials for biomedical applications. For example, glycopeptide supramolecular assemblies, based on ligand-receptor interactions, are able to induce cell spheroids, which is a model 3D cell culture and organoids (Zhou et al., 2018[122]). Adaptively interacting with cell adhesion proteins, the glycopeptide assemblies simultaneously reduce cell adhesion to the substratum and enhance intercellular interactions. By adjusting intercellular force, a key factor in the process of morphogenesis, these assemblies have the ability to generate cell spheroids rapidly. This type of ligand-receptor interaction on cell surface also enables medical imaging of bacterial infections. Based on vancomycin (Van) binding to the D-Ala-D-Ala moiety in the Gram-positive bacterial cell walls, Liu el al. designed a Van based self-assembling peptide derivative for imaging bacteria *in vivo* (Yang et al., 2017[101]). Labeled by a fluorophore and an isotope, it can provide the probes for imaging of deep infection sites. 

Noncovalent interactions between nucleic acids and peptides or nucleopeptides are well-developed for designing biomaterials that find applications in gene delivery (Wang et al., 2019[85]). For example, the integration of nucleobases and peptides to bind with DNA represents a rational approach to create soft biomaterials (Du et al., 2017[16]). These designed nucleopeptides can bind with ssDNA, interact with plasmid DNA and hairpin DNA, promising potential applications in DNA delivery. Apart from DNA delivery, noncovalent interactions between nucleic acids and peptides or nucleopeptides also have found applications for targeted therapy. In this case, a nucleopeptide interacts with ATP or ADP, forming assemblies, sequestering ATP over ADP, and slowing down the efflux of anticancer drugs (Wang et al., 2018[80]). In a related study, a D-peptide derivative spontaneously forms nanoparticles, which interact with RNA to form membraneless condensates in the nucleolus after clathrin-mediated endocytosed by tumor cells (Wang et al., 2019[81]). The assemblies near nucleolus are able to induce DNA damage and cell death as a result.

Secondly, pH is a highly versatile trigger for modulating noncovalent interactions because the strength of hydrogen bonding is pH-dependent. For example, Lin et al. (2012[60]) reported a pH-responsive injectable hydrogel. Using histidine residues as molecular switches, they generated a viscoelastic liquid near pH 5.5. After being injected, at physiological pH above 6.5, the liquid became self- supporting hydrogels that act tissue scaffolds for regenerative medical applications. The acidic environment present in much of the tumor parenchyma also acts as a trigger for drug release. A pH-sensitive peptide amphiphile developed by Moyer et al. (2014[66]) demonstrates reversible disassembly at low pH. These peptide amphiphiles self-assemble to form nanofibers, which can encapsulate drugs. Altered by external signals, noncovalent interactions can change biomaterial properties like mechanical properties, globally or locally. There is growing evidence showing that mechanical properties of the cellular environment such as rigidity and external stresses play an important role in regulating many cellular functions like migration, contraction, proliferation, and differentiation. 

Other physical changes are temperature or light irradiation. Comparing to temperature, light offers a more precise local control for modulating cellular process. Compared with traditional photochemistry to form covalent bonds, light controlled noncovalent interactions have real-time reversible mechanical tuning ability, which supports more complex cell behaviors (Hörner et al., 2019[38]; Wu et al., 2018[96]).

### Enzymatic supramolecular biomaterials

The noncovalent and enzymatic nature of EISA leads to the use of enzymes, which is ubiquitous in biological systems, to trigger self-assembly to generate different molecular ensembles. This approach, directly connecting the transformation of biomaterials with numerous physiological and pathological processes, has received considerable exploration in the past two decades. By discussing the following representative examples, we briefly introduce the applications of enzymatic supramolecular biomaterials in (i) tissue engineering, (ii) cancer therapy, (iii) drug delivery, (iv) imaging, and (v) other applications.

#### Tissue engineering

**Figure 2:**
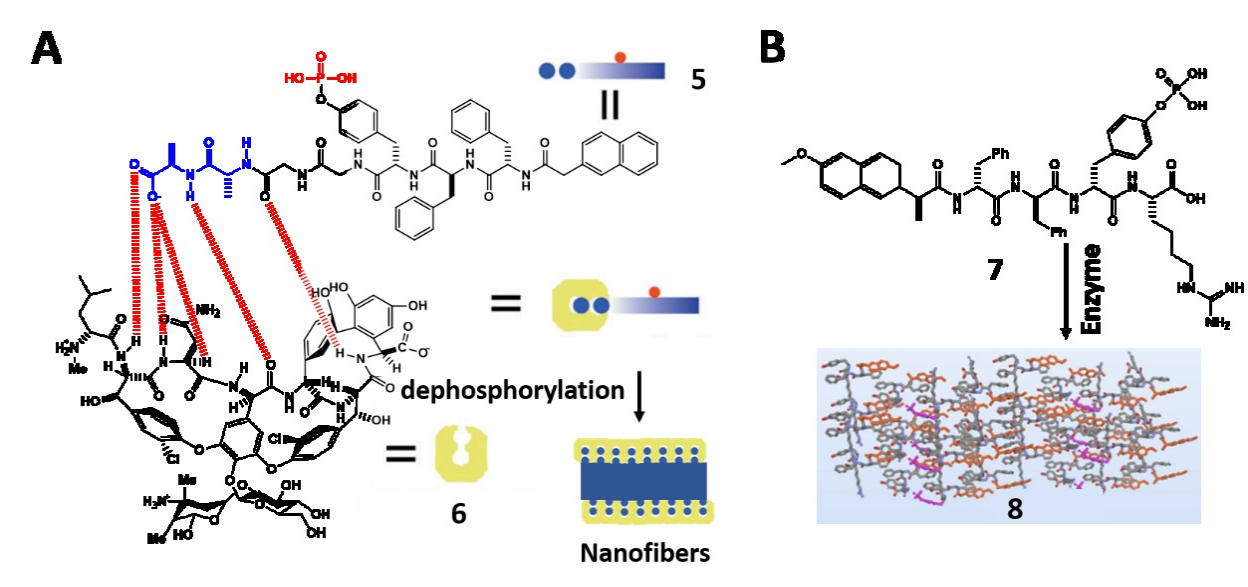
(A) Illustration of 5 that undergoes dephosphorylation to form 6 and self-assembles to form nanofibers. Adapted from Wang et al., 2019c (https://doi.org/10.1002/anie.201812998). Copyright © 2018 by Wiley Inc. (B) The conversion from 7 to 8 by enzymes. Adapted from Feng et al., 2018b (https://doi.org/10.1021/jacs.8b10542). Copyright © 2018 by American Chemical Society.

Extracellular matrix (ECM), a three-dimensional network of extracellular biomolecules, plays important roles in cell adhesion, proliferation, differentiation, and communication (Clark and Brugge, 1995[13]; Engler et al., 2006[18]; Lauffenburger and Horwitz, 1996[47]; Sternlicht and Werb, 2001[74]). The highly dynamic feature of ECM coincides with EISA, which provides an insight into developing ECM mimicking biomaterials. A recent study reports the design of supramolecular phosphoglycopeptides (sPGPs) assemblies that transform 2D cell sheets into 3D cell spheroids, mimicking the functions of ECM (Wang et al., 2017[88]). Based on the ligand-receptor interactions between phosphopeptides (**5**) and glycopeptides (**6**), these two components self-assemble to form nanoparticles, which transform into nanofibers upon dephosphorylation in the cell milieu (Figure 2A(Fig. 2); References in Figure 2: Feng et al., 2018[23]; Wang et al., 2019[84]). The co-culture of cancer cells (Saos-2) and stromal cells (HS-5) in different ratios is able to tailor the level of ALP expression, which controls the morphological transformation of the two-components assemblies for enabling cell morphogenesis or for inducing cell death (Wang et al., 2019[84]). In this case, the supramolecular assemblies act as a context-dependent signal for controlling cell fates in the co-culture that mimics tumor microenvironment. This insight leads to the development of a homotypic precursor that achieves spatiotemporal control of noncovalent interactions for mimicking ECM dynamics (Wang et al., 2019[85]). In a different study reported by Qi et al. (2018[69]), incorporating proangiogenic drug into ECM mimicking hydrogel facilitates vasculogenesis because the densely glycosylated hydrogel serves as a reservoir for the sustainable release of drug and induces capillary morphogenesis both *in vitro* and *in vivo *(Qi et al., 2018[69]).

Besides extracellular biomimetic nanomaterials, supramolecular assemblies are able to mimic intracellular biomacromolecules for enzyme sequestration. For example, a phosphopeptide precursor (**7**) targeting ER undergoes dephosphorylation and forms nanofibers of **8** that sequestrate enzymes enriched in ER (Figure 2B(Fig. 2)) (Feng et al., 2018[23]). A trimethylated phosphopeptide undergoes ALP-instructed intracellular morphological transition to form bundles of artificial filaments inside the cells (Feng et al., 2020[21]). The biomimetic filaments hardly interfere with endogenous compartments, providing insights for intracellular filaments engineering. 

#### Cancer therapy

Cancer is the second leading cause of death in the 21^st^ century. Although various treatments have been developed, a large number of patients suffer from cancer progression and recurrence. Recent studies have revealed the complexity of cancer, such as tumor heterogeneity, tumor microenvironment, multidrug resistance (MDR), abnormal metabolism, and epigenetic alteration (Hanahan and Weinberg, 2011[31]). These findings not only explain the failure of chemotherapies in certain cancers to some extent, but also demand new strategies to improve treatment efficacy. For example, connecting taurine to D-peptides via ester bonds boosts their cellular uptake, which is able to counteract the increased efflux pump and the decreased drug absorption caused by MDR (Zhou et al., 2015[123], 2018[120]). Integrating drugs with peptides that are responsive to enzymes upregulated on or inside cancer cells is a useful approach to stabilize some prodrugs until they reach the target sites. Furin-controlled Arg-Val-Arg-Arg-Cys(StBu)-Lys(taxol)-2-cyanobenzothiazole (CBT-Taxol) condensation (Yuan et al., 2015[109]), ALP-instructed self-assembly of anti-cancer peptide NapG^D^F^D^FpYSV (Gao et al., 2019[26]), and CES-guided release of coumarin (Zhang et al., 2019[112]) are a few representative examples of this approach.

**Figure 3:**
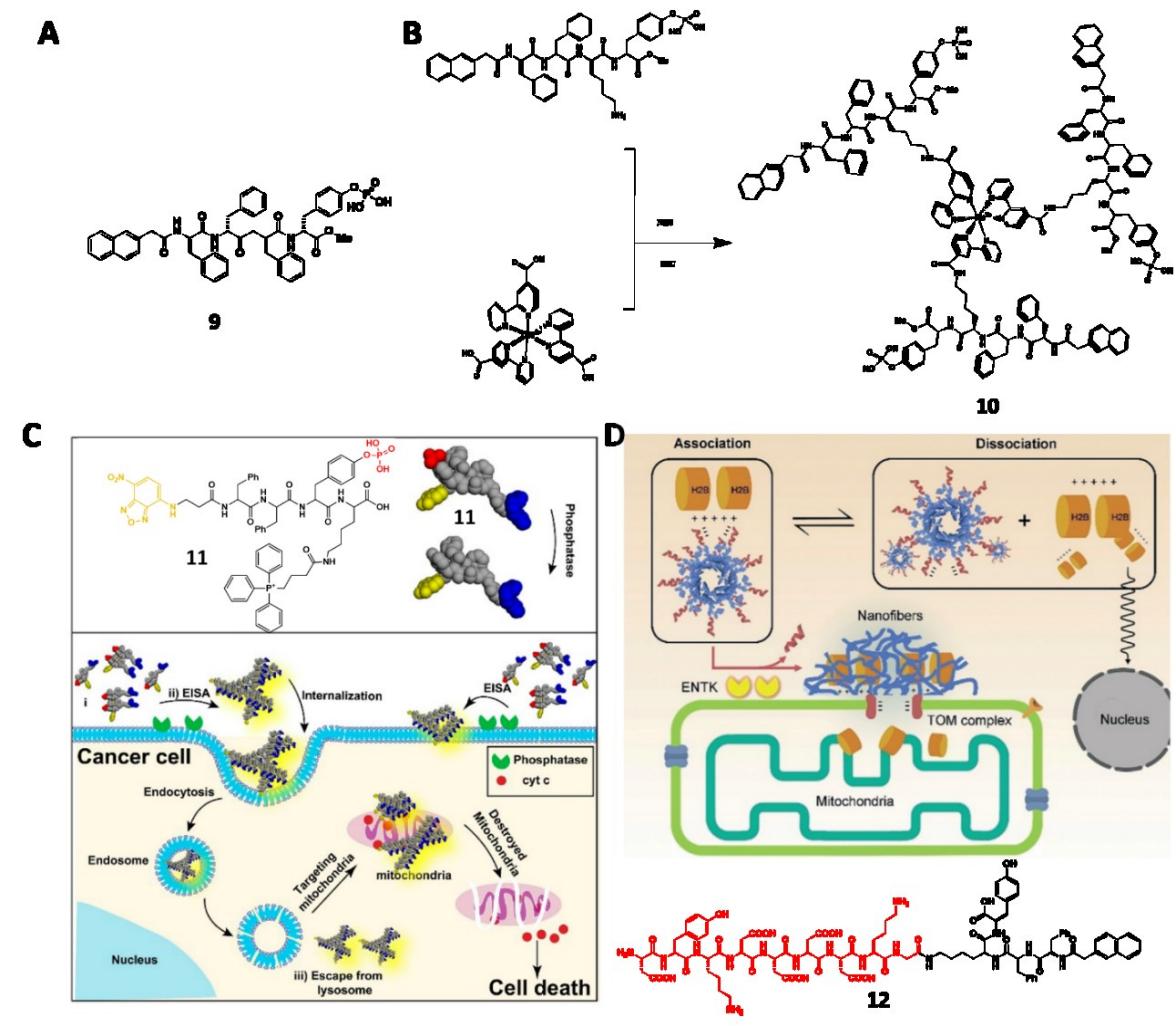
(A) Structure of 9. (B) Structure of 10. (C) Schematic illustration of 11 to target mitochondria of cancer cells and induce cell death. Adapted from Wang et al., 2016a (https://pubs.acs.org/doi/10.1021/jacs.6b09783). Copyright © 2016 by American Chemical Society. (D) Schematic illustration of 12 trafficking H2B to mitochondria. Adapted from He et al., 2020a (https://doi.org/10.26434/chemrxiv.11362508.v1). Copyright © 2020 by Wiley Inc.

Besides making up important parts in prodrugs, the supramolecular assemblies of small molecules are emerging as anticancer drug candidates (Kuang et al., 2014[44]; Kuang and Xu, 2013[46]; Yang et al., 2007[108]). The nanofibers of peptide derivatives formed pericellularly or intracellularly can induce cell death, cytoskeletal rearrangement, and compromised membrane integrity (Li et al., 2018[50]). Specifically, a self-assembling motif (e.g. Nap-FF) is conjugated with an enzyme-responsive (ALP, CES, ENTK or β-galactosidase) substrate to generate a precursor. Upon encountering cancer cells that overexpress these enzymes, the precursor transforms to the hydrogelator via enzymatic bond cleavage, followed by self-assembling process to form nanofibers. By tuning the self-assembling abilities, these nanofibrils can be either innocuous or cytotoxic, exhibiting a molecular dynamic continuum for cancer therapy (Kuang et al, 2014[44]; Feng et al., 2017[20]). For example, a C-terminal methylated phosphopeptide (**9**) is a nontoxic precursor at its optimal concentration. However, **9** induces cell stress and shows strong synergism with NF-κB, verified by adding BAY 11-7085 (BAY) to make the cells sensitive to stress (Figure 3A(Fig. 3); References in Figure 3: He et al., 2020[32]; Wang et al., 2016[82]) (Zhou et al., 2018[121]). A CES cleavable peptide precursor exhibits similar synergism with cisplatin for inhibiting drug-resistant ovarian cancer cells (Li et al., 2015[52]). 

Being the substrates of the overexpressed ALP in some cancer cell lines (e.g., HeLa and Saos-2), several phosphopeptide precursors are able to form toxic nanofibrils on or inside the cancer cells, causing membrane insertion (Du et al., 2019[15]), efficient and selective cell removal (Kuang et al., 2017[45]), cancer cell inhibition (Feng et al., 2019[19], 2020[24]; Wang et al., 2016[83], 2019[87]), or cell rupture (Li et al., 2017[49]). As reported by Zhang et al., a ruthenium-based phosphopeptide trimer (**10**) that favors fast and complete dephosphorylation by glycosylphosphatidylinositol-anchored placental alkaline phosphatase (GPI-anchored PLAP) in lipid raft (Figure 3B(Fig. 3)) enables the cell rupture. Specifically, the assemblies form patches on HeLa cells, resulting in reinforced focal adhesion (FA) on filopodia. The opposite direction of cell migration generates mechanical stress that eventually causes cell rupture. 

Aside from targeting an enzyme or a protein, cell type-specific, dual-targeting nanomedicines offer a better chance for successful targeting cancer cells without inducing acquired drug resistance. Common combinations of dual targets include enzymes and receptors (Wang et al., 2019[92]), enzymes and ligands (Wang et al., 2016[82]), two different enzymes (Zheng et al., 2019[117]), and simply two drugs with different targets (Mang et al., 2019[63]). The dual-targeting concept is highly useful in designing compounds to control cell fates (Zhou et al., 2017[125]), including targeting downregulation in cancer cells (Feng et al., 2017[25]).

Owing to the precise spatiotemporal control of EISA, targeting subcellular organelles or subcellular proteins with EISA is an emerging field in cancer therapy (Feng et al., 2018[22]; He et al., 2018[36], 2020[33]). Integrating extracellular EISA with the mitochondrial targeting ability of triphenyl phosphonium, the peptide derivative (**11**) induce mitochondrial dysfunction and the release of cytochrome c, therefore killing the cancer cells (Figure 3C(Fig. 3)) (Wang et al., 2016[82]). Incorporating ENTK to induce perimitochondrial self-assembly directly delivers chloramphenicol (CLRP) into mitochondria, selectively sensitizing and killing cancer cells (He et al., 2020[33]). A phosphopeptide bearing AVPI segment, which is the antagonistic motif to the inhibitors of apoptotic proteins (IAPs), sequestrates IAPs and selectively induce apoptosis of cancer cells (He et al., 2020[34]). Containing Flag-tag and acting as the substrate of ENTK, a negatively charged peptide derivative (**12**) traffics a nuclear protein, histone protein (H2B) to mitochondria (Figure 3D(Fig. 3)). Being the first example of EISA to traffic endogenous proteins, this work provides insights to understand inter-organelle cross talk (He et al., 2020[32]).

#### Drug delivery

In recent years, many efforts have focused on incorporating enzymatic supramolecular hydrogels in drug delivery systems because it is easy to synthesize the hydrogels that are biocompatible and versatile. Aiming for desired therapeutic effects, common goals of drug delivery are (i) improving pharmacokinetic properties, (ii) involving active targeting, and (iii) achieving sustained release. 

Due to the hydrophobic and unspecific nature of many anticancer drugs, clinical administration of these chemotherapies often suffers from low solubility, poor bioavailability, and severe side effects. Commercially available formulations address these problems by encapsulating drugs into liposomes, such as Doxil, or proteins, such as Abraxane. Alternatively, the enzyme-responsive feature of EISA provides another route by selective targeting of enzymes overexpressed on/in cancer cells. In particular, the morphology of precursor changes upon enzymatic cleavage, which results in self-assembly to form drug-encapsulating hydrogels with enhanced cellular internalization and thus improved drug efficacy. For example, a self-delivery hydrogel of LND-GFFpY, reported by Wu et al. (2019[94]), exhibits a 560-fold increase in solubility compared with lonidamine (LND) alone, as well as selective killing of cancer cells. Similarly, a drug-peptide amphiphile comprising a p53-activating peptide ligand, PMI, reported by Yang et al., enhances cellular uptake and achieves nuclear accumulation (Liang et al., 2020[57]). Besides, the CES modified silicon nanoparticles, which respond specifically to CES substrates, can form nanogels around the particles. The unique core-shell structure serves as a nanocarrier for doxorubicin (DOX) (Wu et al., 2018[93]).

In addition to better pharmacokinetics, supramolecular hydrogels, due to their slow biodegradation, function as local depots for sustained release of drug. For instance, two MMP-9 responsive peptide amphiphiles, which form spherical aggregates in water, undergo morphological transition to form fibers encapsulating DOX. As reported by Kalafatovic et al. (2016[41]), the fibrous networks serve as depots at the invasive border characterized by MMP-9 overexpression Choosing the substrates responsive to different enzymes is able to finely tune the gelation location. In a study reported by Cheng et al. (2019[12]), a cathepsin B (CTSB) responsive prodrug forms intracellular nanofibers for preventing local tumor recurrence after surgery. Intracellular ALP-responsive co-assembly of dexamethasone (Dex) phosphate and a hydrogelator precursor Nap-Phe-Phe-Tyr(H_2_PO_3_)-OH boosts the anti-inflammation efficacy of Dex, as shown by Tang et al. (2017[75]). They also reported similar efficacy enhancement when Dex is covalently conjugated to a homotypic precursor (Tang et al., 2018[76]), or replaced by other drugs like etoposide (Kiran et al., 2018[43]). 

**Figure 4:**
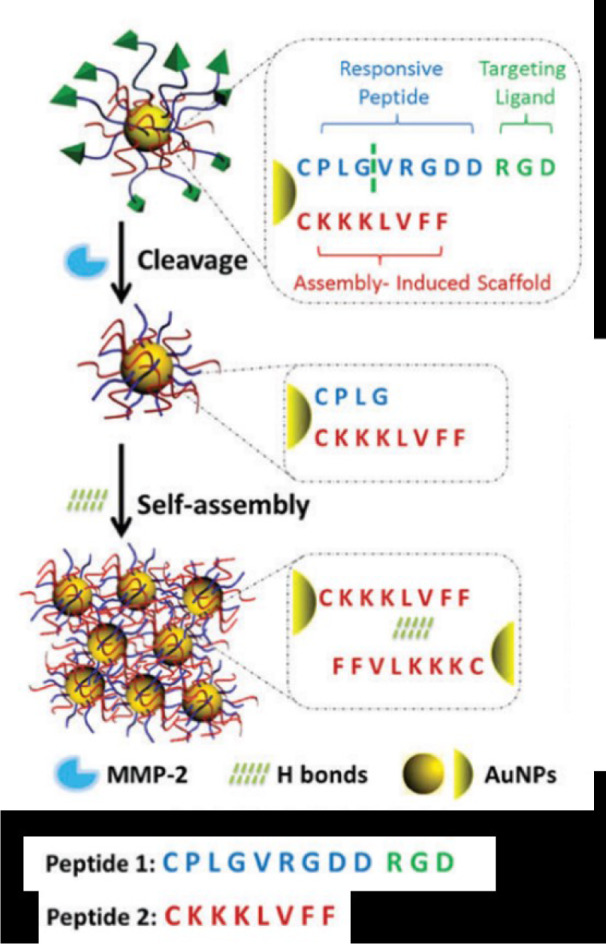
Schematic illustration of MMP-2-triggered self-assembly of gold nanoparticles. Adapted from Hu et al., 2017 (https://doi.org/10.1039/C7TB01268C). Copyright © 2017 by Royal Society of Chemistry.

Another strategy is to use enzyme-responsive gold nanoparticles to enhance photothermal therapy (PTT) efficacy. Hu et al. (2017[39]) conjugated gold nanoparticles with two types of peptide segments on the surface, one is an MMP-2 substrate peptide (CPLGVRGDDRGD), and the other is a self-assembling peptide (CKKKLVFF). Being cut by MMP-2, the strengthened H-bonding between intermolecular self-assembling peptides leads to the aggregation of nanoparticles and red shift in localized surface plasmon resonance (LSPR), thus enhanced PTT efficiency (Figure 4(Fig. 4)) (Hu et al., 2017[39]). A subsequent study by Wang et al. shows that ALP-responsive gold nanoparticles exhibit similar effects (Yang et al., 2018[103]).

#### Imaging

**Figure 5:**
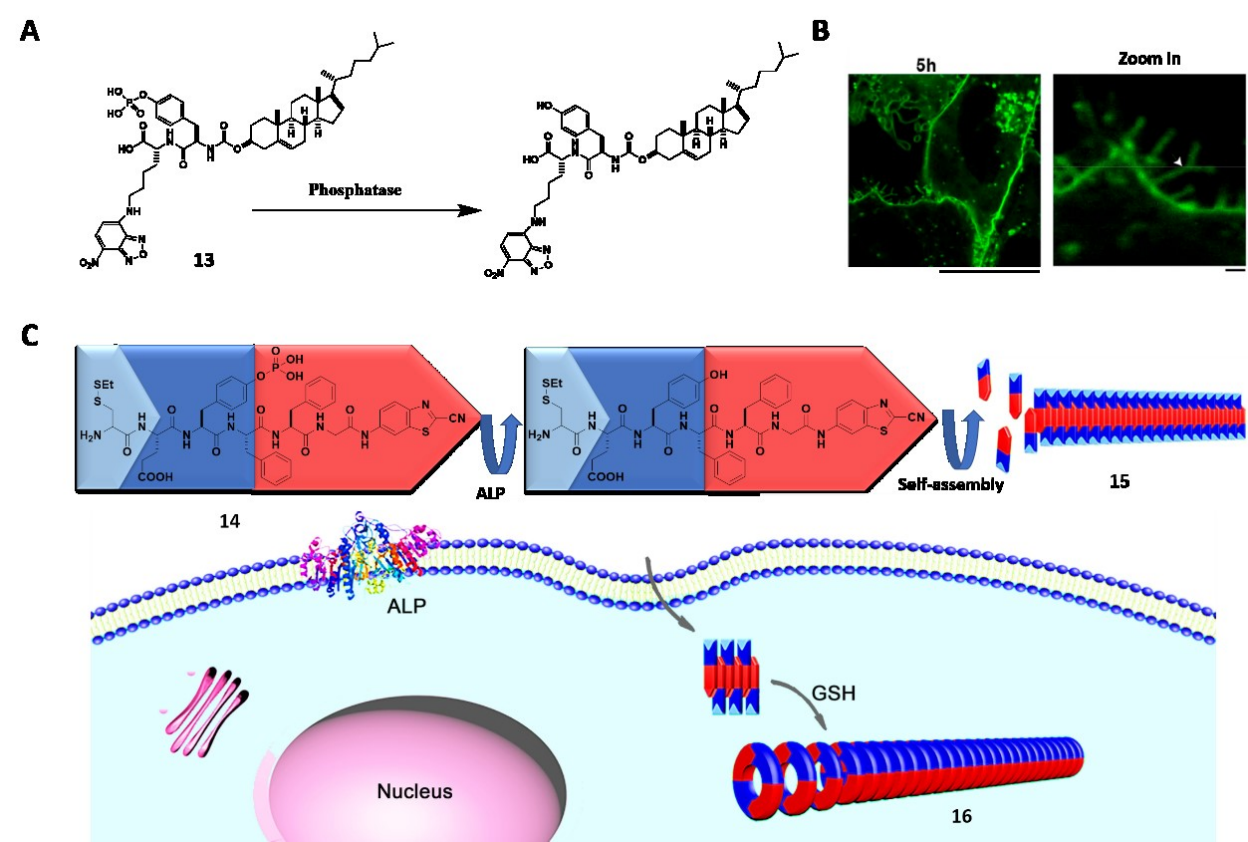
(A) Dephosphorylation of 13. (B) CLSM images of Saos-2 cells treated with 13 (25 µM) for 5 h. Scale bar: 1 µm. Adapted from Wang et al., 2018a (https://doi.org/10.1021/jacs.7b13307). Copyright © 2018 by American Chemical Society. (C) Schematic illustration of 14 that undergoes extracellular ALP-instructed EISA and intracellular GSH-induced condensation generating nanofibers 15 and 16 with different orders of assembly. Adapted from Zheng et al., 2016a (https://doi.org/10.1021/jacs.6b06903). Copyright © 2016 by American Chemical Society.

Being *in situ* and noninvasive, medical imaging techniques such as magnetic resonance imaging (MRI), near-infrared (NIR) fluorescence, computed tomography (CT), positron tomography (PET) and photoacoustic (PA) imaging lead to the development of molecular imaging in the past two decades. The tunability and biocompatibility of EISA allow aberrant levels of enzymes to catalyze the accumulation of imaging agents *in situ* to obtain accurate and precise imaging. Pioneered by Liang et al. (2010[58]), furin-guided CBT condensation initiates self-assembly process to form nanoparticles with dense imaging agents, facilitating MicroPET imaging (Wang et al., 2019[78]; Liu et al., 2015[62]), fluorescence (Li et al., 2019[55]), and phosphorescence imaging (Li et al., 2018[51]) in different cases. As reported by Liang et al., ALP-instructed self-assembly generates nanofibers for enhanced CT (Zheng et al., 2016[119]) and MRI (Dong et al., 2017[14]). Spatiotemporal tuning allows the assemblies to form either extracellularly for cell delineation (Zhao et al., 2017[116]), or intracellularly for imaging (Wu et al., 2018[95]; Wang et al., 2019[90]). Acting on precursors that are responsive to other significant enzymes in physiologic processes, EISA can monitor membrane dynamics. For example, the conjugation of cholesterol with phosphopeptide generates **13** to target lipid raft (Figure 5A(Fig. 5); References in Figure 5: Wang et al., 2018[79]; Zheng et al., 2016[118]). After dephosphorylation by ALP within the lipid raft, the probe selectively assembles in plasma membrane, precisely showing membrane dynamics for an extended period (Figure 5B(Fig. 5)) (Wang et al., 2018[79]). EISA also finds applications in monitoring other dynamic processes or significant components, such as autophagy (Lin et al., 2017[60][61]), Caspas-1 (Li et al., 2016[54]), CTSB (Ni et al., 2019[67]), and fibroblast activation protein-α (FAP-α) (Zhao et al., 2019[115]).

The dual targeting strategy, that is, the use of dual targeting motif (either ligands or enzymes), also applies in molecular imaging (Chen et al., 2019[9]; Hai et al., 2019[30]; An et al., 2020[3]). As reported by Zheng et al. (2016[118]), a precursor (**14**) is able to differentiate the extra- and intracellular environment based on the dual targeting motifs that react with both extracellular ALP and intracellular glutathione (GSH), generating different orders of assemblies (**15** and **16**) (Figure 5C(Fig. 5)). It is also a common strategy to use multiple imaging agents in the precursor. Such a combination can provide comprehensive information to the question of interest, as evidenced by the synergism of NIR and MRI for *in vivo* imaging, as elegantly demonstrated by Yan et al. (2019[100]). Another approach is to incorporate molecular imaging with therapeutic agents to conduct diagnosis and therapy at the same time. The conjugation of drugs with imaging agents provides rich opportunities to develop theranostics (Yuan et al., 2019[110]) that improve imaging process and drug efficacy (Zhang et al., 2018[111]; Ji et al., 2018[40]).

#### Other applications

Apart from a large number of applications for cancer diagnosis and treatment, biomaterials based on noncovalent interactions also play important roles in neuronal development (Mei et al., 2015[65]), antibody production (Wang et al., 2019[91]), and would healing (Li et al., 2019[53]).

## Conclusion and Outlook

Noncovalent interactions, being fundamental in nature, dictate various biological processes. While the tunability and versatility of noncovalent interactions are well-established for generating supramolecular biomaterials as potential biomedicine, the precise control of the reversibility of noncovalent interactions is not only a unique advantage, but also an unmet challenge. The complex and dynamic cellular environment changes noncovalent interactions constantly, making it hard to predict the behavior of supramolecular biomaterials *in vivo*. Various enzymes may also deactivate biomaterials before they reach effective sites. Besides, supramolecular biomaterials require a minimum concentration to assemble, which is hard to maintain under several elimination pathways *in vivo*. Despite several aforementioned examples of supramolecular assemblies in biomedicine, the study of their applications is just beginning and much of the study remains to be carried out. Therefore, unravelling the mechanism of actions, and achieving spatiotemporal control *in vivo* are ultimate goals in developing biomedical applications of supramolecular biomaterials, such as modulating cellular processes and controlling cell fate. To address the unresolved challenges requires the cooperation from chemists, biologists, pharmacists, and engineers. This interdisciplinary research field continues to progress (Wang et al., 2020[89]; Yang et al., 2020[102]; Kim et al., 2020[42]; Xu et al., 2020[98]; Li et al., 2020[56]; Cheng et al., 2019[10][11]; Roy et al., 2020[71]) and to result in interesting discoveries for the years to come.

## Conflict of interest

The authors declare that they have no conflict of interest.
